# Effects of different peritoneal dialysis solutions on serum lipid levels and lipid profile in end-stage chronic kidney disease patients undergoing peritoneal dialysis

**DOI:** 10.1186/s12882-025-04417-4

**Published:** 2025-08-23

**Authors:** Gokhan Aydin, Cevat Topal, Kamil Konur, Oguzkan Ilmaz

**Affiliations:** 1 Faculty of Medicine, Division of Gastroenterology, Department of Internal Medicine, Giresun University, Giresun, Turkey; 2 Medical Park Trabzon Hospital, Division of Nefrology, Department of Internal Medicine, Trabzon, Turkey; 3 Department of Internal Medicine, Recep Tayyip Erdogan University, Rize, Turkey; 4 School of Medicine, Giresun University, Giresun, Turkey; 5 Faculty of Medicine, Department of Internal Medicine, Division of Gastroenterology Giresun, University of Giresun, Giresun, 28000 Turkey

**Keywords:** Peritoneal dialysis, Dialysate glucose load, Dyslipidemia, Lipid profile, HDL cholesterol

## Abstract

Atherosclerotic cardiovascular diseases are the leading causes of morbidity and mortality in patients undergoing peritoneal dialysis and hemodialysis due to end-stage renal disease. Dyslipidemia is a major contributing factor to the development of atherosclerotic cardiovascular diseases, and its prevention is recommended to reduce cardiovascular risk. Several studies have demonstrated the presence of dyslipidemia in patients with chronic kidney disease, and peritoneal dialysis patients have been observed to have a more atherogenic lipid profile compared to those receiving hemodialysis. One of the primary reasons for this is thought to be the absorption of glucose from peritoneal dialysis solutions. In this study, we aimed to assess dyslipidemia in peritoneal dialysis patients and investigate the relationship between glucose absorption from dialysis solutions and lipid profile alterations. A total of 73 patients followed up in the nephrology outpatient clinic were included. Patients’ lipid profiles, the types of dialysis solutions used, and their compositions were statistically analyzed. A statistically significant inverse correlation was found between the amount of glucose absorbed during peritoneal dialysis and HDL cholesterol levels, which was observed both in the overall peritoneal dialysis patient group and in the subgroup undergoing automated peritoneal dialysis (APD). However, no statistically significant relationship was found between glucose absorption and other lipid parameters. Our findings suggest that the glucose content of peritoneal dialysis solutions alone does not play a major role in the development of dyslipidemia when considering all atherogenic lipid parameters. Therefore, dyslipidemia in peritoneal dialysis patients should be evaluated by considering all contributing factors, including treatment-related effects and, in particular, the structural and functional characteristics of the peritoneal membrane.

## Introduction

Chronic kidney disease (CKD) is defined by persistent abnormalities in kidney function or structure with significant health consequences [1]. CKD is characterized as a persistent abnormality in kidney structure or function lasting more than three months (e.g., glomerular filtration rate [GFR] < 60 mL/min/1.73 m² or ≥ 30 mg albuminuria per 24 h) and affects 8–16% of the global population [2]. In the United States alone, CKD affects more than 20 million individuals, with over 500,000 suffering from end-stage renal disease (ESRD) [3]. By 2040, CKD is projected to be the fifth leading cause of years of life lost worldwide [4]. The most common causes of CKD are diabetes and hypertension. CKD is a major global health concern and an independent risk factor for cardiovascular disease, cognitive dysfunction, hospitalization, and all-cause mortality [5]. As CKD progresses to ESRD, patients face significantly increased risks of death and require renal replacement therapy (RRT), most commonly hemodialysis or peritoneal dialysis (PD) [6].

PD offers several advantages such as flexibility, improved quality of life, and cost-effectiveness [7, 8]. Currently, more than 3.5 million people worldwide, including approximately 540,000 in the U.S., receive maintenance dialysis, with a five-year survival rate of about 40% [9]. Advances in PD have reduced complications and allowed longer use, while insights into peritoneal transport mechanisms have opened new avenues for therapeutic optimization [10]. Nevertheless, cardiovascular risk and lipid metabolism disturbances remain major clinical concerns in PD patients.

Even at mild levels of kidney failure, independent of traditional risk factors, the risk of cardiovascular disease is increased, reaching its peak in ESRD patients requiring dialysis [11]. One of the most critical pathophysiological mechanisms contributing to cardiovascular disease in CKD patients is the widespread and potentially accelerated formation of atherosclerotic plaques due to hyperlipidemia, uremic toxins, inflammation, oxidative stress, and endothelial dysfunction [12]. Various aspects of serum lipid profiles and HDL metabolism, including their structure and function, can be significantly altered in patients with nephrotic-range proteinuria or CKD. These abnormalities contribute to cardiovascular complications, foam cell formation, atherosclerosis, and/or glomerulosclerosis in affected patients [13]. While dyslipidemia plays a significant role in cardiovascular disease development in CKD patients, the composition of dialysis solutions directly influences lipid metabolism in PD patients.

Since its integration into standard care, PD therapy has evolved to enhance biocompatibility and improve both local peritoneal and systemic outcomes. New PD solutions, advanced manufacturing techniques, and alternative osmotic agents have been utilized to improve peritoneal survival and patient experience [14]. Dyslipidemia is a common metabolic complication in PD patients and has traditionally been assessed primarily in terms of cardiovascular risk [15]. The amount of glucose absorbed during peritoneal dialysis varies depending on the type of dialysis and the glucose concentration of the solution, contributing to hyperglycemia and insulin resistance, which in turn increase VLDL and LDL levels [16, 17]. It has been reported that a 10 g/day increase in peritoneal glucose intake is associated with a 0.145 mmol/L rise in serum cholesterol levels [18]. Furthermore, PD patients have been shown to exhibit a more atherogenic lipid profile compared to those on hemodialysis, likely due to continuous glucose absorption and treatment-related factors [19].

This study aims to assess dyslipidemia in PD patients and investigate the relationship between the total glucose intake from dialysis solutions and dyslipidemia. By doing so, we will examine the impact of dialysis-related glucose intake on the development of a more atherogenic lipid profile in PD patients compared to hemodialysis. We believe this study will provide valuable guidance in treatment planning for patients with chronic kidney disease.

## Materials and methods

A total of 93 patients who had started PD and were being followed and treated at the same center were included in the study. Twenty patients were excluded from the study because they did not meet the study criteria. Among these patients, 11 were using lipid-lowering medication, and 9 had started peritoneal dialysis within the last three months, making them ineligible for inclusion. The exclusion criteria for the study were as follows: use of lipid-lowering drugs within the last three months, presence of familial dyslipidemia syndrome, undergoing peritoneal dialysis for less than three months, changes in dialysis solutions within the last three months prior to the study, active infections, history of peritonitis in the last six months, and abnormal thyroid function test results.

A total of 73 patients who met the study criteria were included. The personal information of the patients, such as gender and age, current medications, previously diagnosed and existing diseases, duration of peritoneal dialysis, types of dialysis solutions used, and the duration of their usage were recorded. The duration of dialysis and average dwell time were recorded for each patient. However, due to retrospective limitations, detailed peritoneal equilibration test (PET) classifications were not consistently available and thus not included in the analysis. Among the patients, 35 were female, and 38 were male. Fifteen patients were undergoing APD, while 58 were receiving continuous ambulatory peritoneal dialysis (CAPD). Among the included patients, 18 had diabetes mellitus (DM), 63 had hypertension, 17 had type 2 DM, 1 had type 1 DM, 6 had coronary artery disease, 2 had benign prostatic hyperplasia (BPH), 3 had hepatitis B, 1 had hepatitis C, 1 had nephrolithiasis, 2 had ischemic nephropathy, 1 had Alport syndrome, 2 had reflux nephropathy, 3 had chronic glomerulonephritis, 1 had gout, and 2 had polycystic kidney disease. Additionally, 2 patients had a history of hypothyroidism, and 1 had hyperthyroidism, but their FT4 and TSH values were within the normal range during follow-up. None of these patients were using lipid-lowering drugs, and none met the exclusion criteria. The youngest patient was 16 years old, while the oldest was 83 years old, with an average age of 49 years.

The peritoneal dialysis solutions used by the patients were recorded as follows: Dianeal 1.36%, Dianeal 2.27%, Extraneal 7.5%, Physioneal 1.36%, Physioneal 2.27%, PP4 Dianeal 1.26%, PPD Dianeal 2.27%, CAPD2 1.5%, CAPD4 2.3%, and CAPD3 4.25%. Patients used these solutions in various combinations. The total glucose intake of each patient during daily peritoneal dialysis was calculated separately in milligrams (mg), and the total glucose load received by the patients was mathematically determined. Glucose absorption was estimated based on the glucose concentration of the dialysate and the prescribed exchange volumes for each patient. Due to the retrospective nature of the study, individual peritoneal membrane transport characteristics were not available, and direct measurement of glucose absorption (e.g., PET or mass balance) was not performed. To assess lipid parameter levels, which are considered coronary artery risk factors in CKD, total cholesterol, LDL, HDL, and triglycerides were measured using the enzymatic colorimetric method, while lipoprotein(a) was measured using nephelometric analysis (Beckman-Coulter). Blood samples were collected from patients after 12 h of fasting for these parameters. The risk levels of lipid parameters were evaluated based on the Turkish Society of Cardiology Coronary Heart Disease Prevention guideline and the National Cholesterol Education Program Adult Treatment Panel-3 criteria.

The measured lipid profiles (all lipid parameters in mg/dL) and the total glucose intake from peritoneal dialysis (mg/dL) were statistically analyzed. The study aimed to determine lipid profile changes and dyslipidemia in peritoneal dialysis patients, investigate the relationship between glucose intake from dialysis solutions and lipid profiles, and evaluate the association between glucose intake and dyslipidemia. Additionally, lipid profiles of patients undergoing CAPD and APD were separately analyzed and compared.

Data analysis was performed using the SPSS for Windows 11.5 software package. The normality of continuous variable distributions was assessed using the Shapiro-Wilk test. The significance of differences in median values of laboratory measurements between dialysis groups was analyzed using the Mann-Whitney U test. The chi-square test (Pearson’s chi-square test) was used to assess significant differences in the distribution of cases according to laboratory threshold values between dialysis groups. The correlation between continuous variables was examined using Spearman’s correlation test. A multivariate linear regression analysis was performed including glucose load, dialysis duration, and presence of diabetes as potential confounders to assess independent associations with HDL cholesterol levels. Results with a p-value of < 0.05 were considered statistically significant.

## Results

A total of 73 patients undergoing peritoneal dialysis were included in the study. Among them, 15 were undergoing APD, while 58 were on CAPD. The mean total cholesterol level of all cases was 186.95 mg/dL, HDL was 41.00 mg/dL, LDL was 107 mg/dL, triglycerides were 163.99 mg/dL, lipoprotein(a) was 52.93 mg/dL, and the amount of glucose received through dialysis was 170.15 mg/dl (Table 1). The mean baseline total cholesterol level of 186.95 mg/dL was relatively high, aligning with the dyslipidemia frequently observed in patients with end-stage renal disease undergoing peritoneal dialysis.

**Table 1:** Descriptive statistics of lipid profile and dialysate glucose levels in all cases

Variables	Mean	Std. Deviation	Median	Minimum	Maximum
Total Cholesterol (mg/dl)	186.95	46.90	177.00	115.00	312.00
HDL (mg/dl)	41.00	11.40	39.00	20.00	70.00
LDL (mg/dl)	107.32	39.89	102.00	45.00	220.00
Triglyceride (mg/dl)	163.99	77.74	155.00	62.00	559.00
Lipoprotein a (mg/dl)	52.93	48.96	33.70	2.00	219.00
Dialysate Glucose (mg/dl)	170.15	95.18	136.20	54.40	499.40

**Fig. 1:**
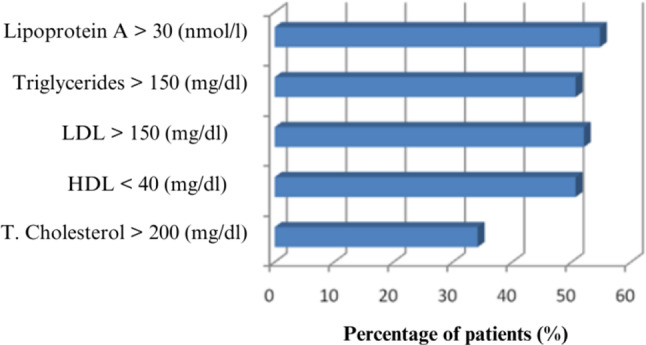
Distribution of cases according to reference values for lipid profile and Lipoprotein(a) measurements

Considering the reference values for lipid levels in terms of coronary artery disease risk, 25 patients (34.2%) had high total cholesterol levels exceeding the risk threshold, 37 patients (57.7%) had low HDL levels, 38 patients (52.1%) had high LDL levels, 37 patients (57.7%) had high triglyceride levels, and 40 patients (54.8%) had high lipoprotein(a) levels (Table 2; Fig. 1.)

**Table 2:** Frequency distribution of lipid profile measurements according to reference values in all cases

Variables	*n* (%)
High Total Cholesterol	25 (34.2)
Low HDL	37 (50.7)
High LDL	38 (52.1)
High Triglyceride	37 (50.7)
High Lipoprotein(a)	40 (54.8)
Total	73 (100)

**Fig. 2:**
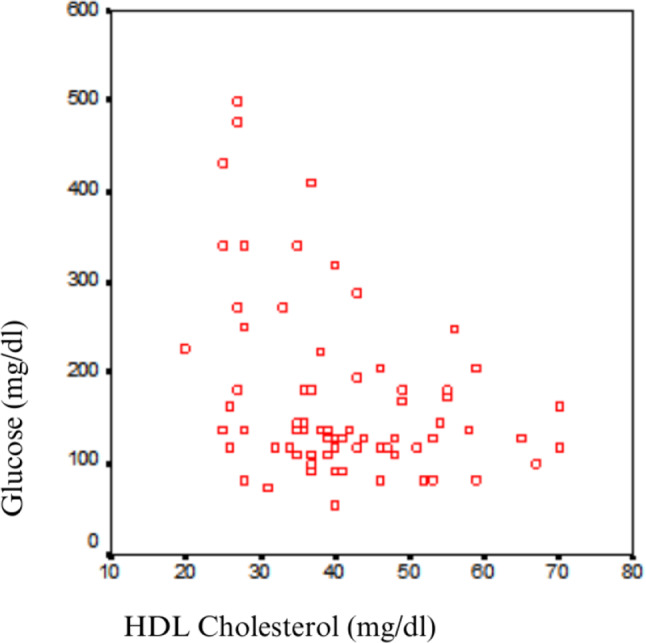
Scatter plot showing the inverse correlation between dialysate glucose load and HDL cholesterol in all PD patients (*r* = -0.293, *p* = 0.012)

There was no statistically significant correlation between glucose levels and total cholesterol, LDL, triglycerides, or lipoprotein(a) (*p* > 0.05). However, a statistically significant inverse correlation was found between glucose and HDL cholesterol (*r* = -0.293, *p* = 0.012). In multivariate analysis (Table 3), glucose load remained a significant independent predictor of HDL cholesterol levels (β = -0.25, *p* = 0.03), while diabetes mellitus and dialysis duration were not significantly associated with HDL cholesterol. In other words, as the glucose level from peritoneal dialysis increased, HDL cholesterol levels decreased (Table 4; Fig. 2).

**Table 3:** Multivariate linear regression analysis for predictors of HDL cholesterol levels

Variables	β Coefficient	95% Confidence Interval	*p*
Glucose Load	-0.25	[-0.47, -0.02]	0.030
Diabetes Mellitus	-0.09	[-0.27, 0.09]	0.345
Dialysis Duration	-0.07	[-0.23, 0.08]	0.362

**Table 4:** Correlation coefficients and significance levels between glucose level and lipid profile measurements in all cases

Variables	*r*	*p*
Total Cholesterol	0,074	0,536
HDL	-0,293	0,012
LDL	0,049	0,679
Triglyceride	0,098	0,412
Lipoprotein (a)	-0,091	0,445

**Fig. 3:**
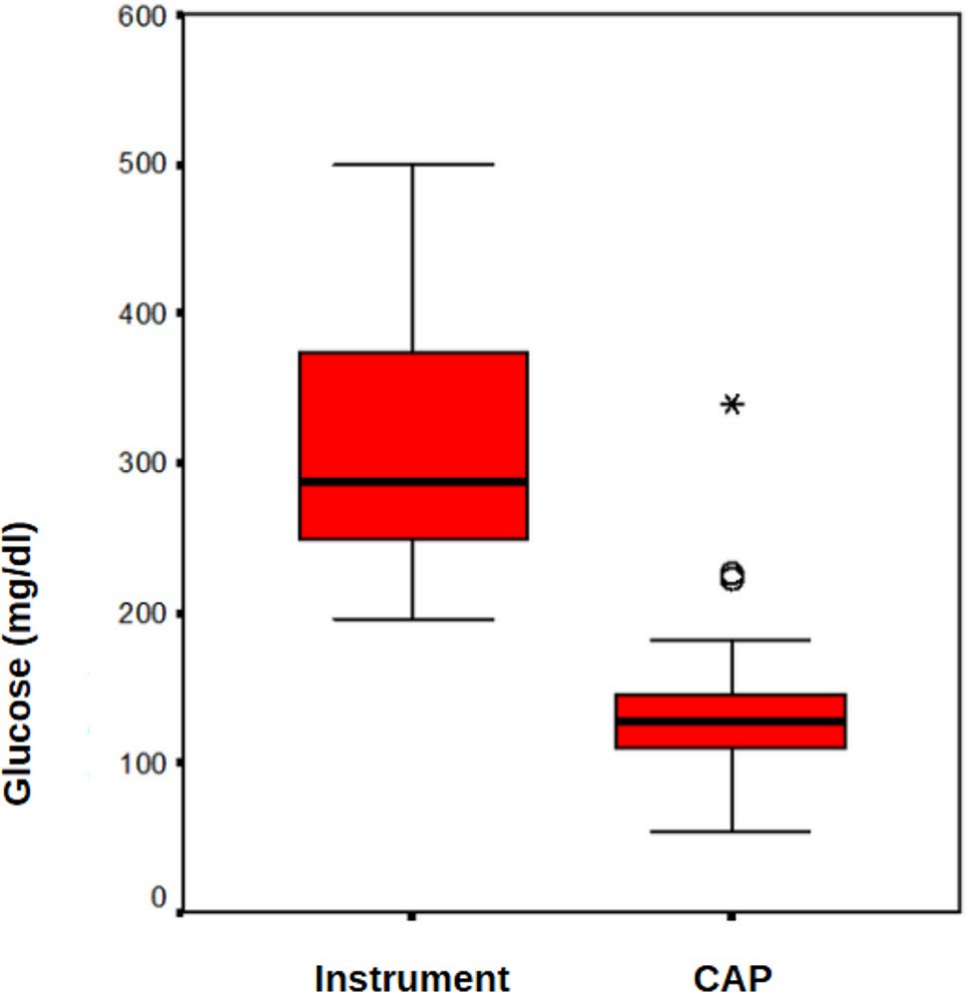
Peritoneal dialysis glucose levels according to dialysis groups

No statistically significant difference was found in lipid profiles between the APD and CAPD groups (*p* > 0.05). However, the median glucose level received through peritoneal dialysis was significantly higher in the APD group compared to the CAPD group (*p* < 0.001) (Table 5; Fig. 3).

**Table 5:** Investigation of lipid profile and glucose levels taken during Dialysis according to instrumental and CAPD Dialysis groups

Variables	Instrument	CAPD	*p*
Total Cholesterol (mg/dl)	180,0 (125,0–283,0)	177,0 (115,0–312,0)	0,672
HDL (mg/dl)	33,0 (25,0–59,0)	40,0 (20,0–70,0)	0,072
LDL (mg/dl)	111,0 (48,0–191,0)	102,0 (45,0–220,0)	0,512
Triglyceride (mg/dl)	188,0 (79,0–292,0)	143,0 (62,0–559,0)	0,246
Lipoprotein a (mg/dl)	37,2 (9,0–136,0)	32,7 (2,0–219,0)	0,417
Glucose (mg/dl)	288,0 (195,1–499,4)	127,0 (54,4–340,0)	< 0,001

There was no statistically significant difference between the APD and CAPD groups in terms of the frequency of high total cholesterol, low HDL, high LDL, high triglycerides, or high lipoprotein(a) levels (*p* > 0.05) (Table 6).

**Table 6:** Distribution of patients according to Dialysis groups in terms of lipid profile measurements and reference values

Variables	Instrument (*n* = 15)	CAP (*n* = 58)	*p*
High Total Cholesterol	6 (%40,0)	19 (%32,8)	0,598
Low HDL	9 (%60,0)	28 (%48,3)	0,418
High LDL	8 (%53,3)	30 (%51,7)	0,911
High Triglyceride	9 (%60,0)	28 (%48,3)	0,418
High Lipoprotein (a)	10 (%66,7)	30 (%51,7)	0,300

**Fig. 4:**
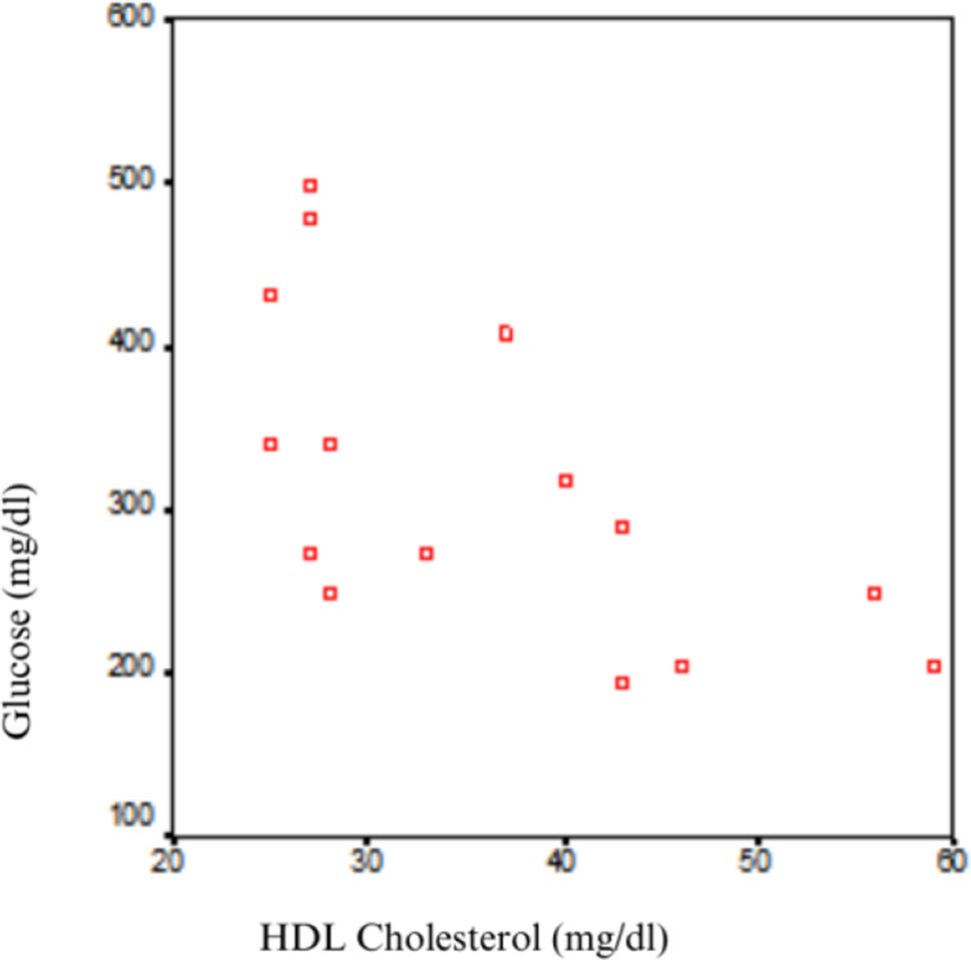
Scatter plot showing the inverse correlation between dialysate glucose load and HDL cholesterol in the APD subgroup (*r* = -0.727, *p* = 0.002; *n* = 15)

Within the APD group, no statistically significant correlation was found between glucose and total cholesterol, LDL, triglycerides, or lipoprotein(a) (*p* > 0.05). However, a statistically significant inverse correlation was found between glucose and HDL cholesterol (*r* = -0.727, *p* = 0.002). In other words, as the glucose intake during peritoneal dialysis increased, HDL cholesterol levels decreased (Table 7; Fig. 4).

**Table 7:** Correlation coefficients and significance levels between glucose level and lipid profile measurements in the instrumental Dialysis group

Variables	*r*	*p*
Total Cholesterol	-0,222	0,427
HDL	-0,727	0,002
LDL	-0,313	0,256
Triglyceride	0,306	0,268
Lipoprotein (a)	-0,111	0,694

Within the CAPD group, no statistically significant correlation was found between glucose and total cholesterol, HDL, LDL, triglycerides, or lipoprotein(a) (*p* > 0.05) (Table 8).

**Table 8:** Correlation coefficients and significance levels between glucose level and lipid profile measurements in CAPD Dialysis group

Variables	*r*	*p*
Total Cholesterol	0,075	0,576
HDL	-0,157	0,240
LDL	0,017	0,899
Triglyceride	-0,008	0,955
Lipoprotein (a)	-0,223	0,092

## Discussion

Many patients today use dialysis solutions with different concentrations and, consequently, varying amounts of glucose. Since glucose can lead to dyslipidemia and other metabolic side effects, glucose-free icodextrin, amino acid-containing, and physiologically pH-balanced new dialysis solutions are being incorporated into treatment plans to improve metabolic control and reduce other side effects associated with high-glucose dialysate use.

Although some previous studies have suggested a positive association between glucose absorption and total cholesterol, our findings did not demonstrate a statistically significant correlation. Only HDL cholesterol was inversely associated with glucose absorption. According to our statistical analyses, a significant portion of the 73 patients undergoing peritoneal dialysis in our clinic exhibited an atherogenic lipid profile in terms of lipid parameters (total cholesterol, LDL, HDL, triglycerides, and lipoprotein a). Among the changes in lipid profiles based on the amount of glucose absorbed during peritoneal dialysis, only HDL cholesterol showed a significant correlation. There was a statistically significant inverse correlation between peritoneal glucose absorption and HDL cholesterol (*r*=-0.293, *p* = 0.012). In other words, as the amount of glucose absorbed during peritoneal dialysis increased, HDL cholesterol levels decreased (Fig. 2). No significant association was found between glucose absorption and other atherogenic lipid parameters (total cholesterol, LDL, triglycerides, and lipoprotein a).

When patients were categorized into APD and CAPD groups, it was observed that the median amount of glucose absorbed during dialysis was significantly higher in the APD group (*p* < 0.001) (Fig. 3). This result was expected, as APD patients undergo dialysis with a larger volume of fluid. However, despite the difference in glucose absorption between the two groups, no statistically significant difference was found in lipid profiles (*p* > 0.05). This lack of statistical significance may partly be attributed to the relatively small number of patients in the APD group, which reduced the statistical power of between-group comparisons. This suggests that neither the amount of glucose absorbed nor the type of peritoneal dialysis is directly linked to lipid profiles. No statistically significant difference was found between the APD and CAPD groups in terms of the prevalence of high total cholesterol, low HDL, high LDL, high triglycerides, and high lipoprotein (a) (*p* > 0.05).

When the APD and CAPD groups were analyzed separately, no statistically significant correlation was found between glucose absorption and any lipid parameters (total cholesterol, LDL, HDL, triglycerides, lipoprotein a) in CAPD patients (*p* > 0.05). Although the inverse correlation between glucose load and HDL cholesterol was statistically significant in the APD subgroup (*r* = -0.727, *p* = 0.002), this subgroup included only 15 patients. Therefore, the findings should be interpreted with caution and cannot establish a temporal or causal relationship. In other words, as glucose absorption increased during APD, HDL cholesterol levels decreased. Even after adjusting for dialysis duration and diabetes status, glucose load was independently associated with lower HDL levels in multivariate analysis. However, due to the observational and cross-sectional nature of the study, these findings should be interpreted with caution and do not establish a causal relationship. No statistically significant correlation was found between glucose absorption and other lipid parameters (*p* > 0.05). This phenomenon in APD may be due to increased transfer of HDL into the dialysis fluid from the peritoneal membrane, leading to decreased serum HDL levels. However, this remains speculative, as dialysate fluid was not analyzed for HDL content in this study. Future studies including direct measurements are warranted.

A relationship has been reported between high glucose concentrations in dialysates and increased mortality [20]. Similarly, high glucose absorption has been associated with dyslipidemia, characterized by increased triglyceride and total cholesterol levels [21, 22]. However, a study by Law et al. found no significant correlation between glucose absorption and lipid profiles after adjusting for confounding variables [23]. Our findings are consistent with the hypothesis that glucose absorption modulates lipid metabolism. Malnutrition and systemic inflammation are dominant factors contributing to cardiovascular disease risk in this population, often outweighing traditional risk factors such as hyperlipidemia [24].

Although various studies have presented differing perspectives, recent long-term studies and our research indicate that the development of dyslipidemia in PD patients is more closely related to the course, treatment, and pathogenesis of CKD rather than the composition of the dialysate used. The statistically significant inverse relationship between glucose absorption and HDL cholesterol in our study may be an important clue in understanding the development of atherosclerosis in PD patients. However, since changes in other lipid parameters besides HDL were not statistically significant, we believe that the glucose content of peritoneal dialysis solutions does not play a major role in the planning of treatment concerning the increase and progression of dyslipidemia when considering all atherogenic lipid parameters.

The fact that LDL cholesterol elevation is primarily responsible for the formation and progression of cholesterol-rich atheromatous plaques in the vascular wall, and that increased glucose absorption during dialysis did not affect LDL cholesterol in our study, may provide another explanation for this phenomenon. While some studies and sources suggest that increased glucose absorption during PD exacerbates the atherogenic lipid profile, our study and recent research do not support this relationship. Therefore, we do not believe that the glucose content of dialysates should be the primary consideration in dialysis solution selection.

## Limitations

The relatively small sample size, particularly in the APD subgroup (*n* = 15), limits the statistical power and generalizability of our findings; therefore, these results should be interpreted with caution. Moreover, the unequal distribution between the APD and CAPD groups may have limited the ability to detect statistically significant associations in subgroup analyses. Another limitation is the indirect estimation of glucose absorption without accounting for individual peritoneal membrane transport characteristics or using PET-based measurements. Additionally, residual renal function data were not available for most patients, which is a limitation in assessing confounding influences on lipid metabolism. Furthermore, the multivariate regression model included only three covariates (glucose load, diabetes mellitus, and dialysis duration), and other potential confounders could not be incorporated due to data limitations. Larger multicenter studies with more comprehensive assessments are warranted to validate and expand upon these observations.

## Conclusion

Glucose absorption through PD alone does not appear to be a determining factor in the development and progression of dyslipidemia. The development of dyslipidemia is associated with various factors in the course of chronic kidney disease, particularly with changes related to the peritoneal membrane. Therefore, further research is needed to better understand the mechanisms of dyslipidemia.

## Data Availability

The datasets generated and/or analyzed during the current study are not publicly available due to institutional regulations and patient confidentiality, but are available from the corresponding author on reasonable request.

## Abbreviations

APD: Automated peritoneal dialysis
BPH: Benign prostatic hyperplasia
CAPD: Continuous ambulatory peritoneal dialysis
CKD: Chronic kidney disease
DM: Diabetes mellitus
ESRD: End-stage renal disease
GFR: Glomerular filtration rate
PD: Peritoneal dialysis
LDL: Low-density lipoprotein
HDL: High-density lipoprotein
TG: Triglycerides
